# Genome-Wide Association Study of Soybean Germplasm Derived From Canadian × Chinese Crosses to Mine for Novel Alleles to Improve Seed Yield and Seed Quality Traits

**DOI:** 10.3389/fpls.2022.866300

**Published:** 2022-03-28

**Authors:** Chanditha Priyanatha, Davoud Torkamaneh, Istvan Rajcan

**Affiliations:** ^1^Department of Plant Agriculture, University of Guelph, Guelph, ON, Canada; ^2^Département de Phytologie, Université Laval, Québec, QC, Canada; ^3^Institut de Biologie Intégrative et des Systèmes (IBIS), Université Laval, Québec, QC, Canada

**Keywords:** genome-wide association study, exotic soybean germplasm, quantitative trait loci, Canadian soybean, seed yield

## Abstract

Genome-wide association study (GWAS) has emerged in the past decade as a viable tool for identifying beneficial alleles from a genomic diversity panel. In an ongoing effort to improve soybean [*Glycine max* (L.) Merr.], which is the third largest field crop in Canada, a GWAS was conducted to identify novel alleles underlying seed yield and seed quality and agronomic traits. The genomic panel consisted of 200 genotypes including lines derived from several generations of bi-parental crosses between modern Canadian × Chinese cultivars (CD-CH). The genomic diversity panel was field evaluated at two field locations in Ontario in 2019 and 2020. Genotyping-by-sequencing (GBS) was conducted and yielded almost 32 K high-quality SNPs. GWAS was conducted using Fixed and random model Circulating Probability Unification (FarmCPU) model on the following traits: seed yield, seed protein concentration, seed oil concentration, plant height, 100 seed weight, days to maturity, and lodging score that allowed to identify five QTL regions controlling seed yield and seed oil and protein content. A candidate gene search identified a putative gene for each of the three traits. The results of this GWAS study provide insight into potentially valuable genetic resources residing in Chinese modern cultivars that breeders may use to further improve soybean seed yield and seed quality traits.

## Introduction

There has been a growing concern regarding the narrowness of the North American soybean germplasm with its potentially detrimental implications highlighted as a calls-to-action (Gizlice et al., 1993; Kisha et al., 1998; Fu et al., 2007; Iquira et al., 2010; Mikel et al., 2010; Barabaschi et al., 2012). The recurrent use of a small population of modern commercial cultivars in breeding programs has been suggested to have exacerbated this problem (Mikel et al., 2010; Keilwagen et al., 2014). Exotic or under-utilized germplasm has emerged as a desirable source of novel genetic variation that could help breeders overcome these concerns (Sneller et al., 2005; Fox et al., 2015; Wang et al., 2017; Kofsky et al., 2018; Gaire et al., 2020; Kilian et al., 2020). However, the use of exotic germplasm has yet to be widely adopted despite a growing body of literature in support of the use of under-utilized germplasm, as well as occurrences of positive contributions from exotic or under-utilized germplasm sources (Palomeque et al., 2009a, 2009b, 2010; Kim et al., 2011; Rossi et al., 2013; Akpertey et al., 2014; Bellaloui et al., 2017). One concern that has been expressed is the hesitancy by breeders to dilute the genetic gains made in breeding programs by potentially breaking up selection signatures (Grainger and Rajcan, 2014; Grainger et al., 2018); and I. Rajcan, personal communication.

The limited understanding of how to properly evaluate the contributions from exotic parents, especially given the quantitative nature of many desirable traits, as well as the environmental factors that influence plant performance, has prevented the widespread use of exotic germplasm (Palomeque et al., 2009a). To understand the role of environment and properly evaluate soybean lines derived from modern adapted × modern exotic crosses, a bi-parental RIL population derived from high-yielding Canadian cultivar “OAC Millennium” and an modern Chinese cultivar “Heinong 38” was evaluated by Palomeque et al. (2009a). Seven seed yield QTL, of which five were universal, and two that were environment-specific were identified (Satt100, Satt162, Satt277, Sat_126, Satt139-Sat_042, Satt194-SOYGPA, and Satt259-Satt576; Palomeque et al., 2009a). However, in a subsequent study, the authors were unable to validate these seven seed yield QTL in a RIL population derived from Pioneer 9,071; a high-yielding Canadian cultivar; and # 8902 a high-yielding modern Chinese cultivar (Palomeque et al., 2010). The seed yield QTL tagged by Satt162 was also found to be linked to three QTL associated with lodging, 100 seed weight, and number of pods per node each (Palomeque et al., 2009b). The authors reported validating this QTL for lodging (Palomeque et al., 2010). Furthermore, Rossi et al. (2013) evaluated two RIL populations derived from high-yielding Canadian cultivars and modern Chinese cultivars (OAC Millennium × Heinong 38, and Pioneer 9,071 × #8902) in Canada, United States, and China and were able to identify two yield QTL in the first population and one yield QTL in the second population, across all environments. It was also reported that yield QTL co-localized with agronomic trait QTL. It should be highlighted that these studies were conducted using bi-parental populations. GWAS, therefore, could potentially help identify novel QTL associated with seed yield and other agronomic traits, while also facilitating the evaluation of the performance of exotic cultivars in a genomic diversity panel.

GWAS, though a relatively novel tool in the disciplines of plant breeding and molecular biology, has seen widespread adoption in crops such as soybean, sorghum, capsicum, and maize (Morris et al., 2012; Wang et al., 2012; Zhang et al., 2015, 2018; Contreras-Soto et al., 2017; Han et al., 2018). GWAS was reported to have better precision at identifying candidate genes compared to conventional methods such bi-parental QTL mapping (Qi et al., 2014). The effect of population structure, kinship, and the extent of linkage disequilibrium (LD) on GWAS has all been highlighted to reduce its accuracy and efficiency of QTL detection (Street and Ingvarsson, 2010; Weir, 2010; Korte, 2013). However, improvements to GWAS design to address these issues of kinship, population structure, and spurious associations can be made through adjustments to the model, adjustment of False Discovery Rate (FDR), and the use of modified kinship and population structure matrices (Hyun et al., 2008; VanRaden, 2008; Wang et al., 2012; Li et al., 2013; Brzyski et al., 2017). Such modifications to GWAS design, along with more recent advancements in computational tools, allow for more robust detection of significant marker-trait association discovery (Takeuchi et al., 2013; Tang et al., 2016; Kichaev et al., 2017; Qi et al., 2020; Yin et al., 2021).

The objective of this study was to identify novel alleles related to soybean seed yield, seed protein, and seed oil concentration, as well as agronomic traits, in a panel of diverse accessions through GWAS. The panel included modern commercial cultivars developed at the University of Guelph, progeny lines derived from crosses between modern adapted Canadian × modern exotic Chinese cultivars, modern Chinese cultivars developed at the Chinese Academy of Sciences, Heilongjiang Academy of Agricultural Sciences, Jilin Academy of Agricultural Sciences, Liaoning Academy of Agricultural Sciences, and Northeast Agricultural University, as well as other experimental lines developed at the University of Guelph.

## Materials and Methods

### Plant Materials

The diversity panel consisted of 200 genotypes of modern Canadian (CD) cultivars (*n* = 59), modern Chinese (CH) cultivars (*n* = 53), and Canadian × Chinese (CD-CH) progeny lines (*n* = 88) belonging to maturity groups 0, 1, and 2 (Supplementary Table S1). The diversity panel was evaluated in yield trials at the Elora Research Station (43°64′104.4” N; 80°40′567.4′′ W), Elora ON, and Woodstock Research Station (43°08′44.8′′ N 80°47′02.5′′ W), Woodstock ON during 2019 and 2020 field seasons. Two replications were evaluated per environment in a nearest neighbor Randomized Complete Block Design (nn-RCBD) with soybean lines randomly assigned.

Seedling emergence score was recorded for each plot 3 weeks after planting, based on the plot-wise number of plants observed. A scale of 0–10 was used where 0 corresponded to no emergence and 10 corresponding to 100% emergence. Pubescence color, flower color, and leaf morphology were recorded subsequently. Flower color was recorded at the R1 stage (one flower at any node), full maturity date was recorded at R8 stage where 95–100% of pods have turned brown; lodging: scored at maturity on a scale ranging from 1 to 5, where 1 = plants fully upright and 5 = plants fully prostrate; and height: as the distance between the terminal node and the ground, measured in cm (Ernpig and Fehr, 1971). All field observations were recorded on an iPad and exported as an MS Excel file.

Seed quality traits were measured using a Perten Diode Array 7,250 Near Infra-Red Spectroscopy (Springfield, United States) machine following manufacturer’s guidelines. Seeds were screened to remove off-types, dirt, and other impurities. A 100 seed weight was measured with a regular commercial scale. Hilum color, 100 seed weight, plot number, entry numbers, and experiment number were entered into the NIR machine for each entry. NIR results were exported as an excel file and screened for errors. Randomly selected genotypes were re-run to ensure that the readings were consistent. Within the soybean seed quality traits, only the protein and oil concentration (expressed as % on a dry seed basis) and 100 seed weight (g) were retained for analysis. One entry each from Elora 2019 and Woodstock 2019 was removed from analysis due to machine error (Supplementary Table S2).

Analysis of variance of seed yield, seed quality, and agronomic traits was conducted using the PROC GLIMMIX procedure in Statistical Analysis Systems (SAS) version 9.4 (SAS Institute Inc., Cary, NC, United States) for RCBD. The GLIMMIX procedure allows the use of generalized mixed linear model—a standard in agricultural research (Camp et al., 2018). “Genotype,” “environment,” and “genotype-by-environment” were considered fixed effects and “block (environment)” was considered random effect.

Due to the unbalanced number of genotypes in 2019 at Elora and Woodstock, data were sorted by year and environment and separated into three sets: 2019 (with 147 genotypes), 2020 (200 genotypes), and combined years (147 genotypes). Using PROC GLIMMIX procedure, the least squared means (LSMEANS) values were calculated for seed yield, protein concentration, and oil concentration for both combined environments and individual environments. Shapiro–Wilk test was conducted using PROC UNIVARIATE to determine the distribution of residuals. PROC PLOT was used to examine the normality of residual distribution. Homogeneity of error variance was tested by conducting Levene’s test on the absolute residuals.

Comparisons were made between environments, genotypes, and genotypes by environments. Tukey–Kramer multiple comparison test was invoked along with the LINES statement to generate statistically significant differences between comparison groups. CONTRAST statements were used along with ESTIMATE statements to test the statistical differences, if any, between the three different genotypic groups.

### DNA Extraction

Leaf tissue was collected into labeled 10 ml plant-tissue collection tubes. One to two young leaves were collected into each tube. These tubes were then transported on ice back to the Soybean Research Laboratory at the University of Guelph in Guelph, ON. Leaf tissue samples were freeze dried with a Labonco FreeZone^®^ freeze dry system (Savant Moduly, Kansas City, MO, United States) for a period of 24 h and stored at −4°C.

Genomic DNA was extracted from samples of freeze-dried leaf tissue by using NucleoSpin^®^ Plant II DNA extraction kit by Macherey-Nagel following the manufacturer’s guidelines. Extracted DNA samples were spot tested with a NanoDrop 8,000 machine (Thermo Fisher Scientific, Waltham, MA, United States) to check for protein/RNA contamination and to verify the quality of genomic DNA. DNA concentration was established with the QuBit 4 DNA Analyzer (Thermo Fisher Scientific, Waltham, MA, United States) and was standardized to 10 ng/μl. A precise volume of 10 μl was pipetted out to two 96-well semi-skirted PCR plates, which were sent to Plateforme d’analyses génomiques [Institut de Biologie Intégrative et des Systèmes (IBIS)], Université Laval (Quebec, QC, Canada) for Genotyping-by-Sequencing (GBS) and SNP calling.

### Genotyping and SNP Calling

GBS was conducted following the methods and recommendations outlined by Elshire et al. (2011), Sonah et al. (2013), and Torkamaneh et al. (2020a,c). The GBS library was created with *Ape*KI restriction enzyme digestion. A 158 million single-end reads were generated with an Ion Torrent Proton System (Thermo Fisher Scientific Inc., USA). These were processed using the Fast-GBS.v2 pipeline (Torkamaneh et al., 2020c). FASTQ files were demultiplexed, trimmed, and then mapped against the soybean reference genome (Williams82 (Gmax_275_Wm82.a2.v1); Schmutz et al., 2010) with an average success rate of 94.4%. SNPs were identified from the mapped reads and filtered out if (i) they were multi-allelic, (ii) the overall read quality (QUAL) score was <20, (iii) the mapping quality (MQ) score was <30, (iv) read depth was <2, and (v) missing data >80%. Missing data imputation was performed using BEAGLE v5.1 (Browning et al., 2018) following the protocol laid out by Torkamaneh and Belzile (2015).

### Genome-Wide Association Study

GWAS was conducted using the rMVP package in R (Yin et al., 2021) utilizing Fixed and random model Circulating Probability Unification (FarmCPU; Liu et al., 2016) on the following traits: seed yield, seed protein concentration, seed oil concentration, plant height, 100 seed weight, days to maturity, and lodging score. Of the 200 lines included in the original panel, only 192 were included in GWAS (Supplementary Table S1). The genotypes that were excluded were Canadian cultivars and are listed in Supplementary Material. The FarmCPU model uses multiple loci linear mixed model (MLMM) and incorporates multiple markers simultaneously as covariates in a stepwise MLM to partially remove the confounding between testing markers and kinship (Liu et al., 2016). A genomic PCA matrix (P) and a genomic kinship (VanRaden) matrix (K) were used to capture the population structure and relatedness among individuals in the panel (Hyun et al., 2008; VanRaden, 2008; Li et al., 2013). Genomic Association and Prediction Integrated Tool (GAPIT; Lipka et al., 2012) was used to capture the LD decay of the SNP panel (Tang et al., 2016). An adjusted *p* value following methodology outlined by Brzyski et al. (2017) was used to ensure a false discovery rate (FDR) < 0.05 and to establish a significance threshold (Wang et al., 2012; Brzyski et al., 2017).

### Candidate Gene Search

SNP markers significantly associated with a trait identified through GWAS were compared to previously reported markers and genes annotated in SoyBase Genome Browser (http://soybase.org) and NCBI RefSeq database following similar methodology to Zhang et al. (2015) to determine potential candidate genes. A length of 250 kb was added or removed from either end of the significant marker to locate potential regions for comparison based on the LD rate of the current population. In selecting candidate genes, the following criteria was used as: (i) genes of known function in soybean related to the trait under study, (ii) genes with function-known orthologs in Arabidopsis related to the trait under study, and (iii) genes pinpointed by the peak SNPs. Putative candidate genes were subsequently researched in the literature for verification.

## Results

### Phenotypic Analysis

Mean yield across environments was 2,590 ± 727.9 kg/ha, with a range of 126 kg/ha—4,805 kg/ha (Supplementary Table S3). Analysis of variance revealed that genotype and genotype-environment were the main sources of variation, with environment also showing significance, albeit of smaller magnitude (Supplementary Table S4).

The mean protein concentration observed across environments and years was 41.0% ± 1.95% (dry basis) with a range of 34–46.8%. Analysis of variance showed that genotype, environment, and genotype-by-environment effects were all significant at explaining the variation in the observed protein concentration.

The mean oil concentration across all environments was 19.8% ± 1.23% (dry basis), with a range of 14.9–23.1%. For oil concentration, genotype, environment, and genotype-by-environment effects were all significant.

Correlations were calculated to evaluate the relationships between seed yield, protein concentration, oil concentration, seed weight (g), height (cm), days to maturity, emergence score (1–10, %), and lodging score (1–5, %). Yield was found to be positively correlated with height (*r* = 0.47; *p* < 0.0001) and lodging score (*r* = 0.28; <0.0001), emergence sh (*r* = 0.56; *p* < 0.0001). Yield and oil concentration (*r* = −0.12; *p* < 0.0001), as well as yield and protein concentration (*r* = −0.08; *p* = 0.0035) showed significant negative correlations (Supplementary Table S5).

Protein concentration was negatively correlated with yield (*r* = −0.08; 0.0035), oil concentration (r = −0.40; <0.0001), height (*r* = −0.23; *p* < 0.0001), and days to maturity (*r* = −0.15; *p* < 0.0001). A significant positive relationship was observed between protein concentration and seed weight (*r* = 0.18; *p* < 0.0001). Protein concentration was not correlated with emergence nor lodging.

Oil content showed significant negative relationships with seed yield (*r* = −0.12; *p* < 0.0001), protein content (*r* = −0.40; *p* < 0.0001), height (*r* = −0.05; *p* = 0.0411), seed weight (*r* = −0.12; *p* < 0.0001), days to maturity (*r* = −0.26; *p* < 0.0001), and lodging score (*r* = −0.24; *p* < 0.0001). There was a significant positive relationship observed between oil and emergence (*r* = 0.07; *p* = 0.0088).

Correlation analysis between each location-year for seed yield, seed protein content, and seed oil contents revealed that Elora 2019 showed a significant positive relationship with Woodstock 2019 (*r* = 0.46; *p* < 0.0001); however, Elora 2019 was not correlated to either Elora 2020 or Woodstock 2020 for this trait. Yield at Elora 2020 was correlated with yield at both Woodstock 2019 (*r* = 0.17; *p* = 0.0045) and Woodstock 2020 (*r* = 0.16; *p* = 0.0017). For both protein and oil concentration, all environments were found to be correlated with each other (Supplementary Table S6).

### Genotyping and SNP Calling

A total of 158 million single-end reads were generated by GBS using the Ion Torrent Proton system. These reads were then mapped against the soybean reference genome (Williams82 (Gmax_275_Wm82.a2.v1); Schmutz et al., 2010) with an average success rate of 94.4%. From a total of 119,065 SNPs identified from mapping, 31, 931 SNPs remained after filtering as described in M&M. A final number of 27,911 SNP markers with minor allele frequency (MAF) > 0.05 were retained for GWAS.

**Figure 1:**
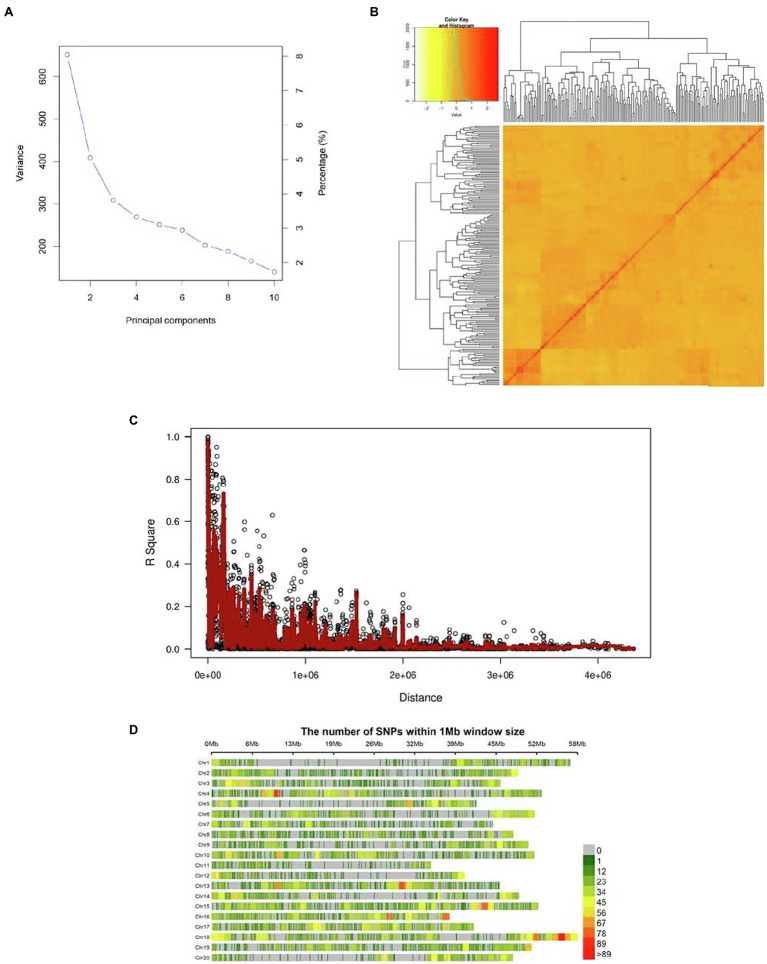
**(A)** PCA (Scree plot) plot depicting the population structure of the 200 soybean genotypes, **(B)** the heat map of the kinship matrix pf 200 soybean genotypes of the current GWAS, **(C)** the genome-wide average LD decay (*R*^2^) of the GWAS panel, and **(D)** genome-wide SNP coverage showing the number of SNPs within 1 Mb window size. Chromosomes appear horizontally with the density of SNPs depicted in the scale shown to the right.

Two major subpopulations, presumably corresponding to the Canadian and Chinese dichotomy, were identified (Figure 1A). The kinship matrix revealed a low level of genetic relatedness among the 200 genotypes (Figure 1B). The LD decay (*r^2^*) of the population was observed to decline to half its maximum value at 250 Kb (Figure 1C). Genomic SNP coverage for the panel of 200 soybean genotypes is depicted in Figure 1D. Evidently, the extent of LD decay varied among the different chromosome regions, resulting in uneven coverage and some regions with no SNPs identified.

### GWAS and Candidate Gene Search

GWAS was carried out using combined environment LSMEANS generated from the analysis reported in above with a total of 27,911 SNP markers used for the following traits: soybean seed yield, seed protein concentration, seed oil concentration, plant height, 100 seed weight, days to maturity, and lodging score using FarmCPU model where P + K values were used as covariates to minimize false discovery rate.

**Figure 2:**
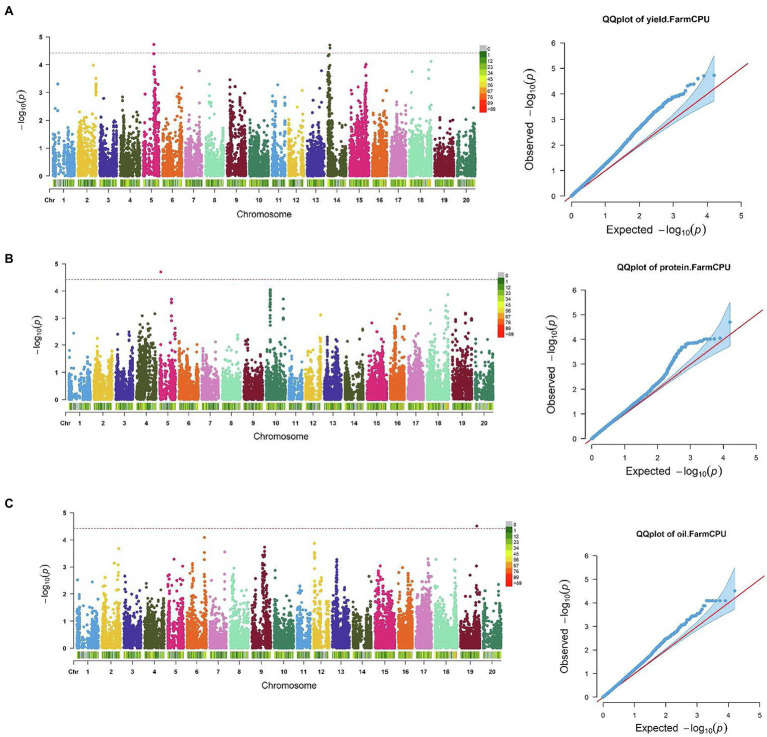
Manhattan plots and corresponding Q-Q plots showing significantly associated SNPs detected in combined environment GWAS analysis for: **(A)** soybean seed yield; **(B)** seed protein; and **(C)** oil concentration. The red horizontal line indicates the significance threshold. Each colored dot represents a SNP.

**Figure 3:**
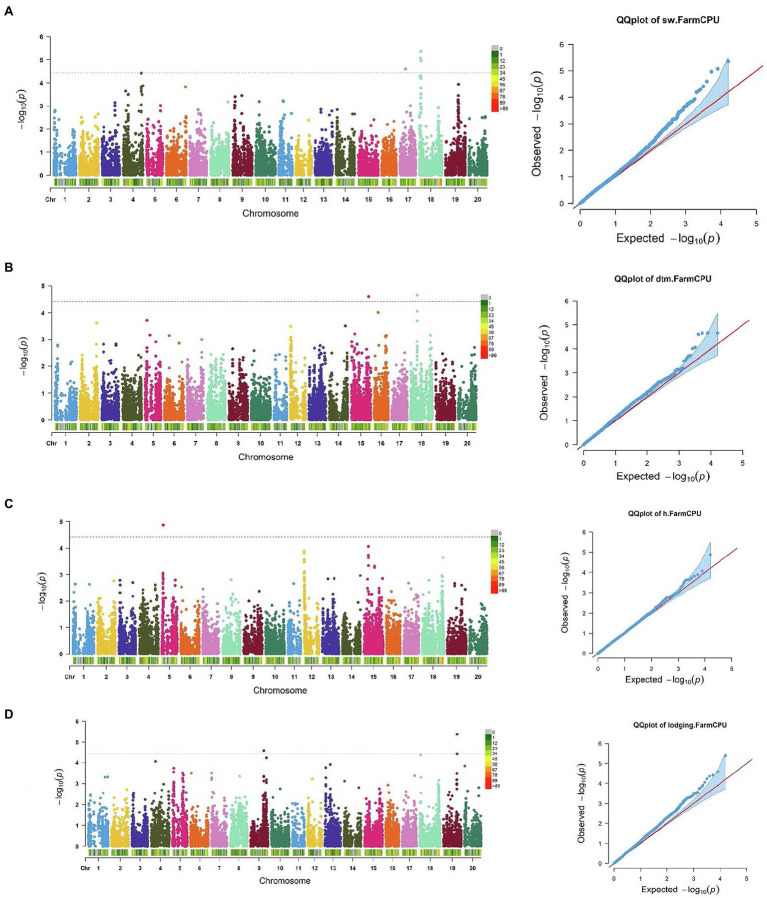
Manhattan plots and corresponding Q-Q plots showing significantly associated SNPs detected in combined environment GWAS analysis for the agronomic traits: **(A)** 100 seed weight; **(B)** days to maturity; **(C)** plant height; and **(D)** lodging score.

SNP markers that were significantly associated with the traits of interest are listed in Tables 1 and 2. The Manhattan plots and the corresponding Q-Q plots for these traits are depicted in Figures 2, 3. In total, 14 significant marker-trait associations were identified. Of these, only the SNPs significantly associated with soybean seed yield (three SNP), seed protein concentration (one SNP), and seed oil concentrations (one SNP) were selected for candidate gene searching. The significant SNP markers associated with the agronomic traits were excluded from candidate gene search due to resource limitations and details of those traits being out-of-scope for the current study. For the agronomic traits, a total of four SNPs were identified for 100 seed weight, two SNPs for days to maturity, two for lodging score, and one SNP for plant height.

**Table 1:** Significant associated genomic regions for soybean seed yield, seed protein, and seed oil concentrations detected in combined-year GWAS analysis.

Trait	Peak SNP ID [Chr_Position(bp)]	Chr	ma	POS	Effect^a^	SE	Value of *p*	Candidate Gene	Role
Seed Yield	S05_27,809,193	5	T	27,809,193	162.9	37.1	1.89E-05	NA^b^	NA
	S14_5,870,227	14	G	5,870,227	−111.2	25.4	1.98E-05	NA	NA
	S14_5,884,688	14	T	5,884,688	−108.8	25.2	2.54E-05	Glyma.14 g072200	Inositol-pentakisphosphate 2-kinase 1
Protein Concentration	S05_3,040,140	05	C	3,040,140	0.86	0.19	1.99E-05	Glyma.05 g03760	Subtilisin/kexin-related serine protease
Oil Concentration	S19_43,240,106	19	C	43,240,106	−0.40	0.09	3.08E-05	Glyma.19 g171000	Zinc finger FYVE domain containing protein

a The effect of the minor allele on the respective trait.

b NA, not available in database.

Chr, chromosome number.

**Table 2:** Significant associated genomic regions for the agronomic traits: 100 seed weight, days to maturity, plant height, and lodging score detected in combined-year GWAS analysis.

Trait	Peak SNP ID [Chr_Position(bp)]	Chr	ma	POS	Effect^a^	SE	Value of *p*
Seed Weight	S17_14,271,552	17	C	14,271,552	−1.27	0.29	2.53E-05
	S18_2,625,222	18	G	2,625,222	−1.12	0.24	8.53E-06
	S18_3,536,348	18	C	3,536,348	−1.03	0.22	4.38E-06
	S18_3,820,958	18	C	3,820,958	−0.85	0.19	1.09E-05
Days to Maturity	S15_46,719,323	15	T	46,719,323	4.87	1.12	2.48E-05
	S18_17,449,562	18	G	17,449,562	6.38	1.46	2.20E-05
Plant Height	S05_4,738,203	5	G	4,738,203	−4.19	0.94	1.35E-05
Lodging Score	S09_38,461,706	9	A	38,461,706	0.12	0.03	2.65E-05
	S19_39,376,171	19	G	39,376,171	0.12	0.03	4.16E-06

a The effect of the minor allele on agronomic trait.

Chr, chromosome number; ma, minor allele; POS, position, and SE, standard error.

The effect magnitudes of the minor allele on seed yield ranged from −111.2 to 162.9 (Table 1; Figure 2). One SNP was significantly associated with seed yield on Chr 5 (S05_27,809,193) while two were detected on Chr 14 (S14_5,870,227 and S14_5,884,688). However, a candidate gene was identified for only S14_5,884,688, with an effect magnitude of −108.8 (Table 1). *Glyma.14 g072200* was reported to encode inositol-pentakisphosphate 2-kinase 1 (*IPK1*) and was identified as a potential candidate gene based on its function and proximity to S14_5,884,688. For seed protein concentration, a single SNP on Chr 5 (S05_3,040,140) was identified as significantly associated, with an effect magnitude of 0.86. *Glyma.05 g03760*, which encodes proprotein convertase subtilisin/kexin, was identified as a potential candidate gene for S05_3,040,140 (Table 1; Figure 2). For seed oil concentration, a single SNP on Chr 19 (S19_43,240,106; Table 1; Figure 2), with an effect magnitude of −0.40, was identified through FarmCPU. Glyma.19 g171000, which encodes zinc finger FYVE domain containing protein, was identified as the potential candidate gene for S19_43,240,106.

Allelic effect for the significant marker-trait associations for seed yield, oil, and protein concentration QTL was also measured. Based on allele frequency, phenotypic data, and effect magnitudes of the minor allele for the significant marker-trait associations identified from FarmCPU, it is likely that both the Canadian and Chinese genotypic groups may have potentially contributed the favorable allele to the seed yield QTL S14_5,870,227, S14_5,884,688, and S05_27,809,193 and the favorable allele for protein concentration QTL (S05_3,040,140) in the CD-CH group (Table 3). The Canadian group was identified as likely to have been the major contributor of the favorable allele for the seed oil QTL (S19_43,240,106) in the CD-CH group (Table 3).

**Table 3:** Distribution of alleles in Canadian, Chinese, and CD-CH germplasm for soybean seed yield, seed oil, and protein concentration QTL.

Trait/QTL	Genotypic Group^1^	Frequency of the favorable allele^2^	Favorable allele
Yield	S14_5,884,688		G
	Canadian	20.34%	
	Chinese	18.87%	
	CD-CH	50.00%	
	S14_5,870,227		A
	Canadian	20.34%	
	Chinese	20.75%	
	CD-CH	50.00%	
	S05_27,809,193		T
	Canadian	8.47%	
	Chinese	5.66%	
	CD-CH	9.09%	
Protein Concentration	S05_3,040,140		A
	Canadian	35.59%	
	Chinese	22.64%	
	CD-CH	43.18%	
Oil Concentration	S19_43,240,106		A
	Canadian	57.63%	
	Chinese	11.32%	
	CD-CH	35.234%	

1 Number of soybean cultivars within each genotypic group that constituted the 200-member GWAS panel: Canadian (*n* = 59), Chinese (*n* = 53), and CD-CH (*n* = 88).

2 Frequency of the favorable allele within each genotypic group. Favorable allele as determined based on estimated SNP effects from FarmCPU.

For 100 seed weight, significant SNP-trait associations were detected in Chr 17 (S17_14,271,552) and Chr 18 (S18_2,625,222, S18_3,536,348, and S18_3,820,958; Table 2; Figure 3). The effect magnitudes of the minor allele ranged from −0.85 to −1.27 for this trait. Chr 18 also contained one of the two significant SNP associations for days to maturity (S18_17,449,562), with the other SNP located on Chr 15 (S15_46,719,323) for days to maturity (Table 2; Figure 3). The effect magnitudes ranged from 4.87 to 6.38 for this trait (Table 2). Only a single significant SNP was identified for plant height on Chr 5 (S05_4,738,203) with an effect of −4.19. Lastly, two SNPs were identified on Chr 9 (S09_38,461,706) and Chr 19 (S19_39,376,171) for lodging, with effect magnitudes of 0.12 (Table 2; Figure 3).

## Discussion

The extent of LD has been reported in literature to be a critical factor in mapping resolution, affecting the number of markers required for adequate coverage of the genome for GWAS (Nordborg et al., 2002; Clark et al., 2007; Weir, 2008; MacKay et al., 2009; Viana et al., 2017). The variation of LD decay observed in different regions of the chromosomes infer that there were potentially missed true trait-QTL associations. Furthermore, the LD decay observed in the current study follows close to values reported in the literature for soybean (Greenspan and Geiger, 2004; Zhang et al., 2015, 2018; Torkamaneh et al., 2020b, 2021). Clark et al. (2007) suggested that roughly one marker per kb was sufficient for genomic coverage for predominantly self-pollinating crops. A total of 28,750 SNP markers would have been required for appropriate SNP coverage for the current study as per Shultz et al. (2006). Since a total of 27,911 SNP markers were retained for GWAS after processing, the number of SNPs retained was deemed adequate (Jorgenson and Witte, 2006; Shultz et al., 2006; Torkamaneh and Belzile, 2015). Genome-wide SNP coverage observed in the current study was low with large gaps (Figure 1D); therefore, better SNP coverage with fewer chromosomal gaps may to help identify more trait-QTL associations in the future. He et al. (2017) provided suggestions on how to improve GWAS for low levels of polymorphisms and shortened LD decay distance. Further refinement could be achieved by using LD block mapping (Bandillo et al., 2015), inclusion of haplotype blocks (Greenspan and Geiger, 2004; Contreras-Soto et al., 2017), SNPLDBs (He et al., 2017), and the inclusion of RILs in the GWAS panel to help maximize the heritability of QTL (Viana et al., 2017). These could all help improve the robustness of trait-QTL associations and increase the rate of detection. Furthermore, Mohammadi et al. (2020) provide additional steps to improve detection of true marker-trait associations through GWAS and validate QTL.

Three putative candidate genes were identified for seed yield, seed protein concentration, and seed oil concentration through GWAS in a panel of Canadian-Chinese soybeans. All the seed quality trait QTL identified in the current study appeared novel. Both Canadian and Chinese germplasm were identified to have contributed potentially beneficial alleles to both seed yield and seed protein QTL in the CD-CH group. This provides further support to the beneficial nature of exotic germplasm. Moreover, the observed allele distribution among the CD-CH group implies further opportunities for increasing seed yield and seed quality traits, especially as indicated for the seed yield QTL identified on chromosome 5. Validation of these QTL in these populations would be necessary in future studies to confirm their effect in these traits. The QTL identified for seed oil concentration was only 1,275 kb away from Pal19, a QTL identified by Smallwood et al. (2017) for palmitic acid. Results of this GWAS, along with the results reported in the previous chapter, lend further credence to the utility of exotic germplasm as a source of novel genetic variety for continued crop improvement. Yield gain, modified seed protein, seed oil profiles, etc., will continue to be focal points for breeders for decades to come (Smallwood, 2015; Zhang et al., 2015; Bruce et al., 2019). Therefore, the identification of these candidate genes and novel putative QTL provides a potential new source of desirable genetics for further study and investigation.

The candidate gene identified for seed yield, *Glyma.14 g072200*, encodes inositol-pentakisphosphate 2-kinase 1, whose expression was reported by Jin et al. (2021) to be downregulated during seed development stage 5 in soybean. Jin et al. (2021) elucidated the effects of mutations in *IPK1* gene on global changes in the gene expression profiles of developing soybean seeds. Though the QTL of large effect is identified, tracking down the causal gene is a tedious and time-consuming task. In addition, a single large-effect QTL often breaks down into multiple, intricately linked QTL of smaller, and sometimes opposite effects on the phenotype (Doerge, 2002; Flint and Mackay, 2009).

For soybean seed protein concentration, *Glyma.05 g03760*, which encodes protein convertase subtilisin/kexin, was identified as a potential candidate gene. This gene was reported to be a close homolog to the *Arabidopsis thaliana* gene AtSBT1.6 (Clarke et al., 2015). The subtilase family proteases are serine peptidases and may be involved in nonselective degradation of proteins, or as proprotein convertases, involved in a range of processes including peptide hormone processing, plant interactions with microorganisms, seed germination, and distribution of stomata (Schaller et al., 2012). Furthermore, Clarke et al. (2015) reported that *Glyma.05 g03760* was identified to be involved in the symbiosome, which is rhizobia enclosed in a plant-derived membrane to form organelle-like structures (Clarke et al., 2015; De La Peña et al., 2018).

The *Glyma.19 g171000* was identified as the candidate gene for seed oil concentration. This putative gene encodes zinc finger FYVE domain containing protein that was identified by Smallwood (2015) as a potential candidate for Pal19 QTL reported in their study. The zinc FYVE finger domain, named after the four proteins Fab1, YOTB/ZK632.12, Vac1, and EEA1, is a highly conserved domain that binds to phosphatidylinositol 3-phosphate that is found on endosomes (Stenmark et al., 1996, 2002). The given location of Pal19 was only 1,275 kb distance away from the position of S19_43,240,106, which makes it quite likely that they co-locate with the same putative gene. In their study, Pal19 was one of the QTL identified for palmitic acid, which suggests that the QTL identified by the current study may co-localize with the same gene. Furthermore, Hyten et al. (2004) also reported identifying a QTL for palmitic acid in the same region. Further investigation could potentially validate the underlying gene responsible for this valuable trait rendering great benefit to future breeders. Though the effect of the alternate allele at S14_5,884,688 had a strong negative effect on seed yield, it is quite likely that a single large-effect QTL could consist of multiple, closely linked QTL of smaller, and sometimes opposite effects on the phenotype as reported in literature (Doerge, 2002; Flint and Mackay, 2009).

To the best of our knowledge, the current study is the first to investigate a genomic panel consisting of modern Canadian, Chinese, and Canadian x Chinese progeny soybean lines in a GWAS design to identify QTL for soybean seed yield, seed oil, and protein concentrations. The results of this study build upon the findings reported by previous authors (Palomeque et al., 2009a,b, 2010; Rossi, 2011; Rossi et al., 2013). The current study was able to identify novel QTL for seed yield, seed oil, and seed protein concentration, as well as agronomic traits. Though the latter were excluded from the candidate gene search, future studies, with the inclusion of these traits along with improved SNP coverage or alternative approaches, such as high-density mapping, could help to overcome the limitations of the current study. In conclusion, the current study contributes to the growing body of literature furthering our understanding of the true potential of exotic germplasm and the genetics underlying seed quality and agronomic traits in soybean.

## Data Availability Statement

The datasets presented in this study can be found in online repositories. The names of the repository/repositories and accession number(s) can be found in the article/Supplementary Material.

## Author Contributions

IR conceptualized, designed and directed the experiments, contributed to the writing, and edited the manuscript. CP conducted the experiments, analyzed, interpreted, and summarized the results, and wrote the manuscript. DT contributed to the GWAS analysis, interpretation of the results, and edited the manuscript. All authors have read and approved the final manuscript.

## Funding

The financial support from the Canadian Agricultural Partnership, Grain Farmers of Ontario, and the Canadian Field Crop Research Alliance.

## Conflict of Interest

The authors declare that the research was conducted in the absence of any commercial or financial relationships that could be construed as a potential conflict of interest.

## Publisher’s Note

All claims expressed in this article are solely those of the authors and do not necessarily represent those of their affiliated organizations, or those of the publisher, the editors and the reviewers. Any product that may be evaluated in this article, or claim that may be made by its manufacturer, is not guaranteed or endorsed by the publisher.
